# Self-Concept in Childhood: The Role of Body Image and Sport Practice

**DOI:** 10.3389/fpsyg.2017.00853

**Published:** 2017-05-24

**Authors:** Santiago Mendo-Lázaro, María I. Polo-del-Río, Diana Amado-Alonso, Damián Iglesias-Gallego, Benito León-del-Barco

**Affiliations:** ^1^Department of Psychology and Anthropology, University of ExtremaduraCáceres, Spain; ^2^Department of Didactics of Music, Plastic and Body Expression, University of ExtremaduraCáceres, Spain

**Keywords:** self-concept, childhood, body satisfaction, body dissatisfaction, organized sport

## Abstract

The purpose of this study was to explore the differences in satisfaction with body image depending on whether the subject practices organized sport or not, as well as the gender of the children. In addition, the study aims to examine the role of body image and the practice of organized sport on the process of building the academic, social, emotional, family and physical dimensions of self-concept in childhood. To do so, a sample of 944 pupils was used. These children were attending primary school in different centers of the Autonomous Community of Extremadura (Spain) and were between 9 and 12 years of age. The main results of the study show that three out of every four children participating in this study were not satisfied with their figure and one out of every five was very dissatisfied. The satisfaction or dissatisfaction with the figure was similar in boys and girls, although it could be appreciated that the ideal body image is partly conditioned by gender stereotypes. The children most satisfied with their body image had a greater academic and physical self-concept. The children that practiced organized sports had a greater physical and emotional self-concept. The children most dissatisfied with their body image and practiced organized sports had a lower family self-concept. All these findings are discussed with reference to previous research literature.

## Introduction

Body image is a complex concept that symbolizes the way in which people perceive themselves and the way they feel and behave in relation to their own body. This also includes social and individual dimensions (Aguado, 2004). In this sense, self-perception of body image is determined by each person’s physical traits, making up a subjective vision of one’s own body, parallel to the objective idea that others have concerning body image (Castañer and Camerino, 2012).

Body image is not constant throughout time, but is rather a dynamic construct that changes over the different stages of the life cycle (Rodríguez and Alvis, 2015) and which is built up both historically and culturally (Raich, 2001). Childhood and pre-adolescence are critical stages in a human being’s development. Pre-adolescence in particular is a crucial stage because of the substantial corporal, cerebral, sexual, emotional and social changes that occur. In girls, this takes place between 9 and 12 years of age, while in boys it occurs between 10 and 13 years of age (Mancilla et al., 2012). People do, however, start to construct their own body image in childhood, to be precise, the first body image for a child is the image of another’s body (Levin, 2008). It is in pre-adolescence when human beings are more sensitive to the start of their dissatisfaction with their own body (De Gracia et al., 2007; Jensen and Steele, 2009; Schore, 2015; Blakely-McClure and Ostrov, 2016). This is because one’s own body image is influenced by the subjective evaluations that one makes, as well as by the social evaluations one receives from others (Dasgupta, 2013; Preckel et al., 2013).

So, from a very early age, people are continually bombarded by information concerning their own body image and the image of others. This generates a certain amount of unease and a desire to look after one’s physical appearance, when faced with the necessity to live up to some “body models” created by a society that extols the “cult of the body” (Merino et al., 2001). Several studies have pointed out that while girls prefer a body image associated with slimness, boys prefer a body image where muscles predominate (Raich, 2004; McArthur et al., 2005; De Gracia et al., 2007; Rice et al., 2016).

Body image is an important problem for most pre-adolescents (Ricciardelli and Yager, 2015). According to Vaquero et al. (2013), approximately 50% of children between 7 and 12 years of age wish to be slimmer. During pre-adolescence, are more sensitive to suffering from greater dissatisfaction with the physical changes that occur during their biological development (Meza and Pompa, 2013), as well as the emotional, cognitive and, in particular, social changes that bring about an increase in the preoccupation concerning their physical appearance (Ramos et al., 2010). In this sense, body dissatisfaction is an important risk factor for mental disorders. (Legey et al., 2016). In so far as an excessive preoccupation and negative evaluations concerning body image dominate in a person’s mind, it can give rise to body image or eating disorders (De Gracia et al., 2007; Trujano et al., 2010; Vaquero et al., 2013).

The importance of the study of dissatisfaction with one’s own body affects the personal aspect as well as other factors, among which self-concept should perhaps be stressed (Raich, 2004; Toro, 2004). Nevertheless, self-concept and its relationship with personal wellbeing is a relevant aspect within psychological research (García et al., 2006). Self-concept is considered to be a multidimensional construct (academic, emotional, physical, social and family dimensions: Shavelson et al., 1976), with reference to the general physical, behavioral and emotional labeling that people attribute to themselves (García et al., 2013). It is a central and decisive factor in the development of the personality and basic to the construction of an individual’s identity, besides being a factor associated with physical and sporting activity (Esnaola et al., 2008; Slutzky and Simpkins, 2009; Guillén and Ramírez, 2011), In fact, it has been related with a healthy lifestyle through the promotion of the practice of sports and physical activity (Murgui and García, 2010). Of the dimensions concerning self-concept, the physical aspect is a wider construct than that of body image. In addition, it is considered to be the factor upon which body satisfaction or dissatisfaction depends, to a great extent, during adolescence (Fernández-Bustos et al., 2015). To be precise, there is a decrease in the physical self-concept during pre-adolescence (Núñez and González-Pineda, 1994; Goñi et al., 2006), which is then reversed in early adolescence (Pastor et al., 2003).

Nevertheless, several studies have concluded that, already in pre-adolescence, those who possess good physical fitness show a higher physical self-concept than those who do not (Mitchell et al., 2012; García et al., 2013). In the same way, those who regularly practice sport have a higher self-concept (Pastor et al., 2006; Reigal et al., 2012), particularly the physical self-concept (Esnaola and Revuelta, 2009). In this sense, Moreno (2003) carried out a study of the influence of multidimensional self-concept on a healthy lifestyle in early adolescence. The conclusion was that adolescents who did physical exercise and led a healthy lifestyle acquired higher scores in sporting competence and social acceptance, as dimensions of self-concept. Similarly, the existence of a favorable sporting climate at home is also related with a higher physical self-concept and with a greater frequency of physical and sporting activity (Revuelta and Esnaola, 2011).

As regards the latter, research into body image and physical activity suggests that the relations between them are complex, since they are influenced by numerous factors (Camacho et al., 2006). It has been shown that physical activity can produce a higher self-perception of one’s physical condition and ability, as well as an increase in one’s positive body image (Contreras et al., 2010; Homan and Tylka, 2014; Rodríguez and Alvis, 2015). It can also mean less vulnerability toward pressure from the dominant beauty canons of the moment (Ruíz de Azúa et al., 2005), psychosocial improvements in cognitive vitality and mood (Biddle et al., 2000; Prakash et al., 2015), a decrease in anxiety levels (Rebar et al., 2015), greater emotional wellbeing and satisfaction with life (Shoup et al., 2008; Iannotti et al., 2009; Ramos et al., 2012), improvements in self-concept and self-esteem (Fox, 2000; Moreno et al., 2007; Babic et al., 2014; Murgui et al., 2016), prevention and reduction in clinical depression (De Mello et al., 2013; Mammen and Faulkner, 2013; Knapen et al., 2015), or an increase in the academic, social, emotional, physical and family dimensions of self-concept (Jiménez-Moral et al., 2013; Murgui et al., 2016).

Nevertheless, it should be pointed out that physical, sporting activity has been related with body dissatisfaction (Esnaola and Revuelta, 2009), as well as with the search for improvements in physical attractiveness (Fernández et al. (2010). Similarly, a study carried out by Rodríguez (2009) concluded that when the physical self-concept is low, doing physical exercise can be a potential risk of suffering from eating disorders.

### The Current Research

Diverse studies have posed the question of whether the pattern and trajectory of body dissatisfaction in adults occurs equally in pre-adolescence, placing the origin of the dissatisfaction in childhood, thus considering the necessity of widening the investigation to this stage (Mancilla et al., 2012). For all these reasons, research during this stage is especially relevant.

Given the association of body image with such factors as the practice of sports and gender, this work considers both factors as possible moderating variables of body image satisfaction. To be precise, we shall study the possible differences in body image satisfaction with respect to whether the subjects practice organized sport or not as well as the gender (boys/girls). The consideration is whether, during childhood, the practice of organized sports can be considered a protective factor against body image dissatisfaction or whether the gender stereotypes are present concerning body image satisfaction.

The main objective of this work is to examine the role of body image and the practice of organized sport on the process of building the academic, social, emotional, family and physical dimensions of self-concept in childhood. The school and family contexts are the major sources of information through which children can develop their self-concept. A novel contribution in this research is to consider a new context, the practice of organized sport. This new source provides information for constructing one’s self-concept through comparison with teammates and one’s own internal comparisons (body image). To be more precise, we shall analyze the differences in the dimensions of self-concept in childhood in accordance with the body image, the practice of organized sport and the interaction between both variables. Although the relationships between self-concept and body satisfaction or practicing sports has been studied (but to a lesser degree in childhood), their joint analysis is a contextual novelty. Given the relation between variables, we believe this to be relevant. The question we ask ourselves is whether the perception of body image and practicing organized sports and their interaction are relevant factors for the self-concept dimensions in childhood.

## Materials and Methods

### Participants

A total of 944 primary school pupils between 9 and 12 years of age (*M* = 10.76; *DT* = 1.11), 548 (58%) boys and 396 (42%) girls, participated in the research. They belonged to different schools within the Autonomous Community of Extremadura (Spain). The sample was selected using a multistage sampling by clusters in the abovementioned course years of primary education.

### Instruments

#### Self-Concept

In order to evaluate the children’s self-concept, we administered the AF-5 Self-concept Form 5 Questionnaire (García and Musitu, 2014), which is based on the theoretical, multidimensional self-concept model of Shavelson et al. (1976), made up of five factors with 6 items each: academic, measuring the perception of the self-efficacy of learning (e.g., “I do school work well”); social, which is related to the significance that the individual’s behavior has for others (e.g., “I make friends easily”); family, concerning the family dynamics and relationships (e.g., “They criticize me a lot at home”); emotional, concerning the most subjective and intimate components (e.g., “I am afraid of some things”); and physical, measuring the fundamental incidence of the individual’s aptitudes and general appearance (e.g., “I look after my body”). The participants must show their degree of conformity or otherwise with a series of statements by answering each item on a scale of 1 to 99, where “1 = I totally disagree” and “99 = I totally agree.”

In the present study, Cronbach’s Alpha (α = 0.85) and Compound Reliability (CR = 0.84) indices show that the AF-5 have an adequate global reliability and Average Variance Extracted (AVE = 0.58). Similarly, the factors of the questionnaire have an adequate reliability and AVE ≥ 0.50 (F1: α = 0.84, CR = 0.83, AVE = 0.58; F2: α = 0.80, CR = 0.80, AVE = 0.53; F3: α = 0.82, CR = 0.79, VME = 0.53; F4: α = 0.78, CR = 0.79, AVE = 0.52; F5: α = 0.79, CR = 0.80, AVE = 0.52). It is important to point out that the stability of the factorial structure of the AF-5 has been tested in numerous studies, proving to be a fairly stable instrument (García and Musitu, 2014).

#### Self-Perception of Current Body Size

Satisfaction or dissatisfaction with the body is normally diagnosed using questionnaires and scales that evaluate worries about weight, body shape or the Body Mass Index. Nevertheless, when the influence of the subjective perception of one’s own body is investigated, silhouettes are more frequently used (Almeida et al., 2012).

The Stunkard Figure Rating Scale was used to assess self and ideal body sizes. The Stunkard scale consists of nine silhouette figures that increase in size from very thin (a value of 1) to very obese (a value of 9) (Stunkard et al., 1983). *Self body size* is the number of the figure selected by participants in response to the prompt “choose the figure that reflects how you think you look.” *Ideal body size* is the number of the figure chosen in response to the prompt “choose your ideal figure.” This scale has good validity and test-retest reliability (Thompson and Altabe, 1991; Smith et al., 1999).

For self body size and ideal body size, dummy variables were created for the underweight, normal weight, overweight and obese body size categories.

##### Body Size Satisfaction

This variable is defined as the difference between one’s perceived self body size and perceived ideal body size. A body size satisfaction was created for each participant by subtracting the number of the figure indicated as the ideal body size from the number of the figure selected as the self body size. Three dummy variables were created for body size satisfaction, based on the difference between self body size and ideal body size: satisfied (self = ideal), dissatisfied (self-ideal = ±1), very dissatisfied (self-ideal ≥ ±2).

#### Organized Sports Practice

The practice of physical or sporting activity was evaluated in terms of organized sports, due to the required commitment and assiduity (Isorna et al., 2014). Organized sport is that which is institutionalized, recognized, practiced following certain rules and regulations, and which is developed on the basis of training and official competitions (Blanco et al., 2006). It is also characterized by a greater sporting motivation, frequency and intensity, known as competitiveness or sporting commitment (Blanco et al., 2006; Isorna et al., 2014; Rial et al., 2014). This variable was measured dichotomously (yes/no).

### Procedure

A protocol was followed so as to ensure that data collection was carried out in a similar way during the entire process. Each center was first of all contacted, asking for a meeting with the headmaster to resolve any doubts concerning the aims, objectives, time and school years that would be involved in the research. After acceptance of the proposal, a meeting was set up with the director of studies and the teachers and a convenient timetable was proposed for administering the tests.

As for ethical norms, the study received approval from the Ethics Committee of the University of Extremadura. All the participants were treated in accordance with the ethical norms of the American Psychological Association as far as consent, confidentiality and anonymity of the answers were concerned. Before carrying out the research study, all the participants were informed of the process they were to follow, emphasizing the fact that their participation was voluntary and that the data would be treated with the utmost confidentiality. In addition, informed consent was obtained in writing from the parents/legal tutors and teachers of the children involved in the study.

Prior to administering the questionnaire collectively at the agreed times, the principal researcher briefly explained the procedure and gave instructions on completing the questionnaires. The questionnaire took between 30 and 40 min to complete, during which time the principal researcher was present at all times to resolve any doubts that may have arisen during the process

### Data Analysis

The factorial structure of the AF-5 was evaluated by means of Cronbach’s Alpha, Compound Reliability and the Summarized Mean Variance. The Chi-Square test was used to establish the variance/invariance of the distribution of the participants by gender, by whether they practiced organized sports or not, and by their satisfaction with their figure. In order to determine the possible effects of satisfaction with the figure, as well as that of practicing organized sports, on self-concept, a multivariate analysis (MANOVA) with a *post hoc* Bonferroni correction for multiple comparisons was used, in which the dependent variables corresponded to the scores in the five subscales of the AF-5 questionnaire, and the independent ones corresponded to the children’s satisfaction with their figure and whether they practiced organized sport. The assumptions of normality, randomization and homoscedasticity were contrasted through the Kolmogorov–Smirnov, Rachas and Levene tests, respectively.

Given that this is a cross-sectional, observational study, no causal or explicative relations are established.

## Results

### Modulating Variables of Satisfaction with Body Image: Organized Sporting Practice and Gender

**Table 1** shows the distribution of the participants with respect to satisfaction with their figure, whether or not they practiced organized sports and gender.

**Table 1:** Distribution of the participants by groups concerning satisfaction with the figure, organized sporting practice, and gender.

	Organized sporting practice				Gender					
Body size satisfaction	Yes		No		Girls		Boys		Total	
	*n*	%	*n*	%	*n*	%	*n*	%	*n*	%
Satisfied	81	26.2	143	22.5	104	26.3	120	21.9%	224	23.7
Dissatisfied	185	59.6	353	55.6	214	54%	324	59.1%	538	57
Very dissatisfied	44	14.2	138	21.8	78	19.7%	104	19%	182	19.3
Total	310	100	634	100	396	100	548	100	944	100

As for satisfaction with the figure, it is worth noting that 76.3% (*n* = 720) of the participants are not satisfied with their figure. To be more exact, 73.8% (*n* = 229) of those who practiced organized sport and 77.4% (*n* = 491) of those who did not practice sport are not satisfied with their figure. As for satisfaction with the figure, it is worth noting that 76.3% (*n* = 720) of the participants, 73.8% of those who practiced organized sports and 77.4% of those who did not, were not satisfied with their figure. In addition, the comparison of the groups of satisfaction with the figure indicates the existence of differences [χ^2^(2) = 10.040, *p* = 0.007] with respect to whether they practiced organized sport or not, in the sense that the percentage of subjects who did not practice sports and were very dissatisfied with their figure (21.8%) is greater (*p* ≤ 0.05) than that of those who did practice sports and were very dissatisfied with their figure (14.24%). The percentage of satisfied and dissatisfied subjects who did and did not practice organized sports were equivalent (*p* > 0.05).

Similarly, with respect to gender, the distribution of the groups of satisfaction with the figure were equivalent [χ^2^(2) = 2.237, *p* = 0.327]. However, if we compare the distribution of girls and boys with respect to their sense of dissatisfaction, that is to say, between those who prefer to be slimmer (55%) or more muscular/athletic (20%), then there are noticeable differences [χ^2^(2) = 8.471, *p* = 0.014]. This is because, although the percentage of girls (56%) and boys (54.3%) who prefer to have a slimmer constitution is equivalent (*p* > 0.05), the percentage of boys (23.2 %) who prefer to have a more athletic constitution is greater (*p* ≤ 0.05) than that of the girls (15%).

### Effect of Body Image and Practicing Organized Sport on Self-Concept

**Table 2** shows the means and standard deviations obtained in the AF-5 questionnaire with respect to the groups of satisfaction with the figure and whether they practiced organized sports or not.

**Table 2:** Mean and standard deviation, satisfaction with the figure and organized sporting practice.

Self-concept AF-5	Body size satisfaction	Organized sporting practice					
		Yes		No		Total	
		*M*	DT	*M*	DT	*M*	DT
Academic	Satisfied	7.71	1.85	7.94	1.90	7.78	1.86
	Dissatisfied	7.31	1.93	7.12	1.86	7.39	1.91
	Very dissatisfied	6.69	1.72	7.04	2.79	6.86	2.28
	Total	7.38	1.91	7.20	2.05	7.32	1.96
Social	Satisfied	7.59	1.61	8.02	1.33	7.72	1.54
	Dissatisfied	7.56	1.49	7.46	1.35	7.53	1.45
	Very dissatisfied	7.37	1.65	7.41	1.71	7.39	1.67
	Total	7.55	1.53	7.59	1.41	7.57	1.50
Emotional	Satisfied	5.46	2.11	5.31	2.17	5.42	2.12
	Dissatisfied	5.43	2.05	5.29	2.12	5.39	2.07
	Very dissatisfied	5.29	2.19	4.33	2.28	4.84	2.27
	Total	5.43	2.07	5.17	2.17	5.35	2.10
Family	Satisfied	8.75	1.24	8.78	1.17	8.76	1.21
	Dissatisfied	8.54	1.66	8.29	1.88	8.46	1.73
	Very dissatisfied	7.09	2.27	8.20	1.91	7.61	2.17
	Total	8.50	1.65	8.40	1.74	8.47	1.68
Physical	Satisfied	6.46	1.36	6.21	1.16	6.38	1.31
	Dissatisfied	5.88	1.56	5.28	1.76	5.69	1.65
	Very dissatisfied	4.58	1.96	3.98	1.86	4.29	1.92
	Total	5.95	1.61	5.35	1.77	5.75	1.68

A multivariate analysis of the variance (MANOVA) was carried out to analyze the effect of the different degrees of satisfaction with the figure and practicing organized sports or not on self-concept (AF-5).

Multivariate analysis of the variance revealed significant multivariate principal effects on satisfaction with the figure [Wilks λ = 0.868, *F*(10,1584) = 11.625, *p* < 0.001, η = 0.068], whether or not organized sport is practiced [Wilks λ = 0.966, *F*(5,792) = 5.586, *p* < 0.001, η = 0.034] and on the interaction between both variables [Wilks λ = 0.971, *F*(10,1584) = 2.318, *p* < 0.001, η = 0.014].

The univariate contrasts demonstrate the existence of a significant principal effect on the degree of satisfaction with the figure in the academic self-concept, *F*(2,834) = 10.522, *p* < 0.001, η = 0.026, family, *F*(2,834) = 11.903, *p* < 0.001, η = 0.029, and physical, *F*(2,834) = 44.806, *p* < 0.001, η = 0.101, there being no significant principal effect in the social self-concept, *F*(2,834) = 3.156, *p* = 0.073, η = 0.008, or emotional, *F*(2,834) = 2.242, *p* = 0.107, η = 0.006. The multiple comparisons carried out with respect to satisfaction with the figure indicate that the greater the satisfaction or the lesser the dissatisfaction with one’s figure, the higher the scores (*p* ≤ 0.05) in academic, family and physical self-concept.

As for the practice of organized sport, a significant principal effect was found in the emotional, *F*(1,834) = 4.178, *p* = 0.041, η = 0.005, and physical self-concepts, *F*(1,834) = 9.829, *p* = 0.002, η = 0.012, in the sense that those who practice organized sport obtained higher scores (*p* ≤ 0.05) in both factors, there being no significant principal effect in the academic, *F*(1,834) = 0.125, *p* = 0.724, η = 0.000, social, *F*(1,834) = 0.694, *p* = 0.405, η = 0.001, or family self-concept, *F*(1,834) = 3.365, *p* = 0.067, η = 0.004.

In addition, the analysis of the interaction between satisfaction with the figure and the practice of organized sports showed the existence of a significant interaction between variables in the family self-concept*, F*(2,834) = 5.388, *p* = 0.005, η = 0.013.

The multiple comparisons carried out to interpret the interaction of both variables indicate that: (1) the differences found between the groups concerning satisfaction with the figure in the family self-concept, are only significant among those who practice organized sport, *F*(2,796) = 15.717, *p* < 0.000, η = 0.038, in the sense that those who are very dissatisfied with their figure and practice organized sport obtain lower scores (*p* ≤ 0.05) in comparison with those who are just dissatisfied or satisfied, there being no significant differences in the family self-concept, *F*(2,796) = 6.478, *p* = 0.093, η = 0.006, with those who do not practice organized sport; (2) of the subjects who are very dissatisfied with their figure, those who do not practice organized sport obtain higher scores in the family self-concept, *F*(1,796) = 16.088, *p* = 0.004, η = 0.010, than those who do practice organized sport.

## Discussion

The purpose of this study was to explore the differences in satisfaction with body image depending on whether the subjects practice organized sport or not, as well as the gender of the children. The study also examines the role of body image and the practice of organized sport on the process of building the academic, social, emotional, family and physical dimensions of self-concept in childhood. In this sense, the discussion of the results and their interpretation has been structured on two basic lines.

### Satisfaction with Body Image, Practicing Organized Sport and Gender

First of all, from the descriptive analysis and the comparison of the percentages of the participants with respect to body satisfaction, sporting practice and gender, the results indicated that, independently of whether they practiced organized sports or not, three out of every four participants in this research were dissatisfied, to a lesser or greater extent, with their figure; while more than half would prefer to be slimmer. In this sense, Vaquero et al. (2013) found that 50% of children between 7 and 12 years of age wished to be slimmer. Nevertheless, the percentage of subjects who were very dissatisfied with their figure was greater among those who did not practice organized sports. This fact, following the line of previous studies on physical, sporting activity and satisfaction with body image (Contreras et al., 2010; Homan and Tylka, 2014; Rodríguez and Alvis, 2015), and in spite of the fact that the relationship between body image and physical activity is influenced by many variables (Camacho et al., 2006), suggests the existence of a relationship between the practice of organized sports and satisfaction with body image. Nevertheless, based on the results obtained, in which only one in every four children who practice organized sport is satisfied with their figure, it cannot be stated that practicing organized sport is a key protection factor against dissatisfaction with body image. It is possible that these results are determined by the esthetic demands of certain sports which cause their participants to have a low perception of their body image or because of a relationship between the practice of sport and the search for a better physical attractiveness, since one of the factors that motivates physical activity is precisely that (Fernández et al., 2010). However, this relation has not been sufficiently investigated for young ages.

As for gender, adolescent girls are generally more dissatisfied with body image than boys (Murawski et al., 2009). In this sense, our results showed similar percentages of girls and boys satisfied, dissatisfied and very dissatisfied with their body, although the percentage of boys who would prefer to be more muscular is greater than that of the girls. This would seem to confirm the existence of dissatisfaction with body image in the stages prior to adolescence (Tatangelo et al., 2016). It would also seem to indicate that, prior to adolescence, the preoccupation with body image affects both genders in a similar way, although the influence of the predominant ideal gender esthetic can already be appreciated (Sweeting, 2008; Ramos et al., 2010).

### The Role of Body Image and Practicing Organized Sport on Self-Concept

Secondly, with respect to the multidimensional analysis of self-concept as regards the degree of satisfaction with body image and the practice of organized physical activity; on the one hand, the results of the study showed that when the children were more satisfied or less dissatisfied with the figure, in general, they obtained higher scores in the academic and physical self-concept. This would seem to confirm the relationship between satisfaction with physical traits and self-concept (Raich, 2004; Toro, 2004; Fernández-Bustos et al., 2015).

On the other hand, the children who practiced organized sports achieved better results in the emotional and physical self-concept. In this sense, there are many studies that have pointed out the fact that the physically active subjects have a better self perception physically (e.g., Cruz-Sánchez et al., 2011; Guillén and Ramírez, 2011; Moreno et al., 2013; Babic et al., 2014; Murgui et al., 2016). However, the relation between practicing sports and emotional self-concept is not so clear, as there are few studies that have come to this conclusion (e.g., Murgui et al., 2016). Nevertheless, the positive relation between a high level of physical activity and the emotional wellbeing of adolescents has been demonstrated (Shoup et al., 2008; Iannotti et al., 2009; Ramos et al., 2012); while a greater emotional self-concept implies better control of both situations and emotions, as well as a greater level of commitment in their daily lives (García and Musitu, 2014). This would suggest that some of the factors that characterize the practice of organized sports, such as a greater frequency and intensity, competitiveness or commitment (Blanco et al., 2006; Isorna et al., 2014), could be decisive in the emotional dimension of self-concept.

As for the results found in the family self-concept from the analysis of the interaction between satisfaction with the figure and the practice of organized sports, the fact that the children who practiced organized sports and were very dissatisfied with their figure obtained lower scores in the family self-concept could be related to the existence of physical-esthetic motivations and expectations (either intrinsic or extrinsic) related with the assiduous nature of the sport. It is with good reason that sport is a social construct, and as such, its practice is influenced by the social values of reference (Coakley, 2015). Such is the situation that, when the expectations, either personal and or family, concerning the benefits that sporting activity should bring to one’s physical appearance do not materialize, perhaps because the said expectations were not very realistic, then they could cause an increase in pressure (both internal and external) on the said physical appearance, setting it up above and beyond other factors such as amusement or social relations, which will also probably interfere with family relations and the motivation for the sporting activity, eventually creating dissatisfaction with body image.

### Limitations

The study is limited by the use of self-report indices and the observational design that, along with the possibility of reverse causality between variables, such as sports practice, body dissatisfaction, and physical self-concept, prevents causal inferences from being made, as well as the absence of control of such variables as the sport practiced, the frequency, the intensity, or the level and category of the organized sport practiced. Thus, in future research on self-concept and sport, the control of the aforementioned variables would be advisable. Similarly, in light of the results obtained, it would be of interest to use the control of competitiveness or sporting commitment as possible variables related with self-concept and satisfaction with body image.

## Conclusion

Finally, the main conclusion that can be extracted from this work are as follows: (1) three out of every four children participating in this study were not satisfied with their figure, and one out of every five was very dissatisfied; (2) although there is a clear relation between practicing sport and a higher satisfaction with body image, in our study, only one out of every four children who practice organized sport are satisfied with their figure and almost 15% are very dissatisfied. These data invite us to reflect on the variables that could be minimizing the effect of practicing sport as a protection factor against dissatisfaction concerning body image; (3) the satisfaction or dissatisfaction with the figure was similar in boys and girls, although it could be appreciated that the ideal body image is partly conditioned by gender stereotypes; (4) the children most satisfied with their body perceive themselves as more capable as students, with a better physical aspect and physical condition; (5) the children who practiced organized sports, they perceived themselves in a better light physically and possess better control over situations and emotions; (6) the children who are very dissatisfied with their figure and practice organized sport perceive themselves as being less involved, participative and integrated in the family context.

All this, given the enormous importance that society today puts on body image, shows the need to pay special attention to children’s body satisfaction, as well as to the causes and degree of dissatisfaction with the body. Although it is important to encourage sporting activity in children because of its many physical benefits and its relation with a healthy lifestyle, as well as satisfaction with body image, it is no less relevant to work on the acceptance of one’s own body image and that of other children as possible modulating variables of self-concept, as this is a decisive and central factor in the development of the personality and essential to the construction of an individual’s identity (Esnaola et al., 2008; Slutzky and Simpkins, 2009; Guillén and Ramírez, 2011). To do so, we believe that, as with school, sporting organizations are an ideal framework from which, at an early age, to encourage the acquisition of realistic attitudes toward one’s own body image, as a condition for understanding and accepting the differences in bodies and their diversity.

## Author Contributions

All authors listed, have made substantial, direct and intellectual contribution to the work, and approved it for publication. SML, BLB: analysis and interpretation of the data. SML, MPR, DAA, DIG, BLB: The conception and design of the work; Drafting the work.

## Conflict of Interest Statement

The authors declare that the research was conducted in the absence of any commercial or financial relationships that could be construed as a potential conflict of interest.
